# Introducing the General Polytomous Diagnosis Modeling Framework

**DOI:** 10.3389/fpsyg.2018.01474

**Published:** 2018-08-22

**Authors:** Jinsong Chen, Jimmy de la Torre

**Affiliations:** ^1^Department of Psychology, Sun Yat-sen University, Guangzhou, China; ^2^Faculty of Education, University of Hong Kong, Pokfulam, Hong Kong

**Keywords:** CDM, polytomous responses, item-splitting, saturated model, MML

## Abstract

Although considerable developments have been added to the cognitive diagnosis modeling literature recently, most have been conducted for dichotomous responses only. This research proposes a general cognitive diagnosis model for polytomous responses—the general polytomous diagnosis model (GPDM), which combines the G-DINA modeling process for dichotomous responses with the item-splitting process for polytomous responses. The polytomous items are specified similar to dichotomous items in the Q-matrix, and the MML estimation is implemented using an EM algorithm. Under the general framework, different saturated forms, and some reduced forms, can be transformed linearly. Model assessment and adjustment under the dichotomous context can be extended to polytomous responses. This simulation study demonstrates the effectiveness of the model when comparing the two response types. The real-data example further illustrates how the proposed model can make a difference in practice.

## Introduction

Cognitive diagnosis models (CDMs) have received increasing attention in educational and psychological measurement. CDMs are used to assess the strengths and weaknesses of test takers across a set of attributes. Diagnostic measurements based on CDMs can provide diagnostic and domain-specific information on finer grain size that can be used for different testing purposes. Recently, considerable developments had been added to the CDM literature. Among others, these included additive models like the *additive* CDM (A-CDM; de la Torre, 2011), the linear logistic model (LLM; Maris, 1999), and the reduced reparameterized unified model (R-RUM; Hartz, 2002; DiBello et al., 2007); highly constrained models like the *deterministic inputs, noisy “and” gate* (DINA; Junker and Sijtsma, 2001) model, and the *deterministic inputs, noisy “or” gate* (DINO; Templin and Henson, 2006) model; and saturated models like the log-linear CDM (LCDM; Henson et al., 2009) and the generalized DINA (G-DINA; de la Torre, 2011) model. Applied researchers are also equipped with models that can accommodate higher-order structure (de la Torre and Douglas, 2004), polytomous attributes (e.g., von Davier, 2008; Chen and de la Torre, 2013), multiple strategies (de la Torre and Douglas, 2008), partial credit (de la Torre, 2010), nominal responses (Templin et al., 2008; Chen and Zhou, 2017, March), and multiple-choice (MC) items (de la Torre, 2009). In terms of model assessment, various methods or procedures are provided for Q-matrix validation or more general model misfit (e.g., de la Torre, 2008; Chen et al., 2013; Chiu, 2013; de la Torre and Chiu, 2015; Chen, 2017). With these developments, one can find a growing number of CDM applications across different educational and psychological areas. Specifically, CDM-based measurement can be found in topics such as math (Tatsuoka, 1990), reading (Jang, 2009; Chen and Chen, 2015, 2016), psychological disorder (Templin and Henson, 2006), and situational judgment (García et al., 2014).

The significance of polytomous responses for diagnostic measurement has been realized for quite a while, with different conceptual models available (e.g., Rupp et al., 2010, p. 98). However, we can only identify three estimable CDM frameworks for polytomous responses up to date (von Davier, 2008; de la Torre, 2010; Ma and de la Torre, 2016). The partial-credit DINA model (de la Torre, 2010) is a direct extension of the highly constrained DINA model. In the general diagnostic model (GDM), von Davier (2008) accommodated polytomous responses based on the idea of the generalized partial credit model (GPCM; Muraki, 1992). The GDM framework for polytomous responses is a linearly additive model and no interaction term is involved (see Equation 2 in p. 290). The sequential CDM for polytomous responses (Ma and de la Torre, 2016) adopted the G-DINA model as the process function. But it required that the item categories were attained sequentially and associated with specific attributes explicitly, which might not be always applicable. Moreover, it is unclear how the monotonicity assumption that the probability of responding in higher category will increase monotonically for examinees with more required attributes can be satisfied. Although polytomous responses can be dichotomized, it is suboptimal, and can result in a dramatic loss of information. What is missing is a general, saturated framework for polytomous responses similar to the G-DINA model or LCDM for dichotomous responses, which can subsume a variety of reduced models.

Without a more general and comprehensive modeling framework for polytomous responses, the power of CDMs cannot be fully appreciated by applied researchers and practitioners across a wide range of areas. This research proposes a general cognitive diagnosis model for polytomous responses, the basic logic of which is to combine the G-DINA modeling process for dichotomous responses with the item-splitting process similar to the graded response modeling (Samejima, 1969). The G-DINA monotonicity constraint that examinees with more attributes will not have lower probability of success (Hong et al., 2016) is also extended to polytomous responses. The resulting model will be called the general polytomous diagnosis model (GPDM), which is saturated, and subsumes various saturated and reduced forms for polytomous and dichotomous responses. The marginal maximum likelihood (MML) estimation is adopted and implemented using an expectation-maximization (EM) algorithm. Although the GPDM will reduce to the G-DINA model for dichotomous responses, the varieties of reduced models within the GPDM exceed those under the G-DINA model, owing to the complexity of polytomous responses. Model assessment and adjustment under the dichotomous context will be also extended to polytomous responses.

## Theoretical framework

### Model formulation

For a diagnostic test with *J* items and *K* dichotomous attributes, there will be *L* = 2^*K*^ attribute patterns and a *J* × *K* Q-matrix. Let $\alpha_{l}={\left(\alpha_{l1},\cdots \;,\alpha_{lK}\right)}^{T}$ denote the attribute vector where *l* = 1, …, *L*, and *q*_*jk*_ is the element in row *j* and column *k* of the Q-matrix. Denote the polytomous responses for item *j* as *X*_*j*_ = *c* with *C*_*j*_ response categories, where *c* = 0, …, *C*_*j*_ – 1. For polytomous responses, one can specify *q*_*jk*_ as 1 if mastery of attribute *k* is required to respond *c* or above on item *j*, and as 0 otherwise for any *c* > 0. If one sets *c* > 1 (e.g., *c* = 2) however, it will result in a loss of information since any response below *c* will not be modeled (e.g., 1 will be treated as 0 when *c* = 2). Note that if one sets *c* = *C*_*j*_ – 1, the polytomous responses reduce to dichotomous responses. For maximal information, this research proposes to specify *q*_*jk*_ as 1 if mastery of attribute *k* is required to respond 1 or above to item *j*, and as 0 otherwise. Stated differently, instead of specifying each item category, one only needs to specify the polytomous items similar to dichotomous items in the Q-matrix. The modeling process will take care of the difference across item categories. Accordingly, the Q-matrix is subjected to same criteria of completeness or identifiability for dichotomous responses (e.g., Köhn and Chiu, 2016; Xu and Zhang, 2016).

For item *j*, irrelevant attributes can be omitted and the required attributes is represented by the reduced attribute vector $\eta_{jh}={\left(\eta_{h1},\cdots \eta_{hg},\cdots \;,\eta_{hG_{j}}\right)}^{T}$, where $h=1,\ldots ,H_{j}=2^{G_{j}}$ and $G_{j}=\sum_{k=1}^{K}q_{jk}$. Similar to the G-DINA model (de la Torre, 2011), the attribute vector α_*l*_ is simplified as the reduced attribute vector **η**_*jh*_, the elements of which correspond to the required attributes in Item *j*. It means that the *L* attribute patterns or latent classes of the whole test are simplified to *H*_*j*_ latent groups in item *j*. The simplification proceeds with the help of Item *j*'s q-vector **q**_*j*_. Note that attributes in the reduced vector should follow the same order as the required attributes in α_*l*_. For a test with four attributes for example, $\alpha_{l}={\left(\alpha_{l1},\alpha_{l2},\alpha_{l3},\alpha_{l4}\right)}^{T}$ and *l* = 1, …, *L* = 16. If only the first and third attributes are required in Item *j*, the q-vector is ${\text{q}}_{j}=\left(1,0,1,0\right)$. Accordingly, *G*_*j*_ = 2 and the reduced vector is $\eta_{jh}={\left(\eta_{h1},\eta_{h2}\right)}^{T}={\left(\alpha_{l1},\alpha_{l3}\right)}^{T}$, with *h* = 1, …, *H*_*j*_ = 4. Working with η_*jh*_ instead of α_*l*_ on Item *j*, one only needs to handle four, instead of 16, latent groups.

For latent variable modeling with polytomous responses, it is convenient to split the polytomous item with *C*_*j*_ response categories into *C*_*j*_ dichotomous sub-items, each of which then can be formulated using models for dichotomous responses. The GPDM splits the item indirectly based on the difference of cumulative probability between response categories. The probability of examinees responding *c*, conditional on reduced attribute vector **η**_*jh*_, is *P*(*X*_*j*_ = *c*|**η**_*jh*_) ≡ *P*_*c*_(**η**_*jh*_), with $\sum_{c=0}^{C_{j}-1}P_{c}\left(\eta_{jh}\right)=1$. The cumulative probability of response in Category *c* or above can be denoted as $P\left(X_{j}\geq c|\eta_{jh}\right)\equiv P_{c}^{*}\left(\eta_{jh}\right)$. Under the graded response approach (Samejima, 1969), the relationship between the conditional and cumulative probabilities is:

(1) $$\begin{array}{r} P_{c}^{*}\left(\eta_{jh}\right)-P_{c+1}^{*}\left(\eta_{jh}\right)=P_{c}\left(\eta_{jh}\right) \end{array}$$

with $P_{0}^{*}\left(\eta_{jh}\right)=1$ and $P_{C_{j}}^{*}\left(\eta_{jh}\right)=0$. For item *j*, the number of independent probabilities in $P_{c}^{*}\left(\eta_{jh}\right)$ and *P*_*c*_(**η**_*jh*_) is the same and equals (*C*_*j*_ − 1)*H*_*j*_. It is more straightforward to formulate the model directly with the conditional probabilities *P*_*c*_(**η**_*jh*_) rather than the cumulative probabilities $P_{c}^{*}\left(\eta_{jh}\right)$.

The monotonicity assumption under the G-DINA model for dichotomous responses (de la Torre, 2011) can be extended to polytomous response. Specifically, the relationship between two attribute vectors **η**_*jh*_ and $\eta_{jh^{\prime }}$ can be defined as **η**_*jh*_ ≥ $\eta_{jh^{\prime }}$ if and only if η_*hg*_ ≥ $\eta_{h^{\prime }g}$ for *g* = 1,…, *G*_*j*_. Equality between the two vectors (i.e., **η**_*jh*_ = $\eta_{jh^{\prime }}$) holds if and only if η_*hg*_ = $\eta_{h^{\prime }g}$ for *g* = 1,…, *G*_*j*_. If **η**_*jh*_ > $\eta_{jh^{\prime }}$, it means that attribute vector **η**_*jh*_ has more required attributes compared to $\eta_{jh^{\prime }}$. In its most general formulation, the GPDM allows $P_{c}^{*}\left(\eta_{jh}\right)<P_{c}^{*}\left(\eta_{jh^{\prime }}\right)$ for **η**_*jh*_ > $\eta_{jh^{\prime }}$. In many CDM applications however, it would be reasonable to impose the monotonicity assumption that $P_{c}^{*}\left(\eta_{jh}\right)\geq P_{c}^{*}\left(\eta_{jh^{\prime }}\right)$ whenever **η**_*jh*_ > $\eta_{jh^{\prime }}$. Namely, the cumulative probability of responding in higher category will increase monotonically for examinees with more required attributes. Note that polytomous items with decreasing monotonicity (i.e., negative items) can be addressed by reversing the items, in case needed.

Using different linking functions, $P_{c}^{}\left(\eta_{jh}\right)$ can be linearly transformed into item effects in different saturated forms, as

(2) $$\begin{array}{rcl} F\left[P_{c}^{}\left(\eta_{jh}\right)\right] & = & \beta_{jc0}+\overset{G_{j}}{\underset{g=1}{\sum }}\beta_{jcg}\eta_{hg}+\overset{G_{j}}{\underset{g^{\prime }>g}{\sum }}\overset{G_{j}-1}{\underset{g=1}{\sum }}\beta_{jcgg^{\prime }}\eta_{hg}\eta_{hg^{\prime }}\ldots \\ +\beta_{jc12\ldots G_{j}}\overset{G_{j}}{\underset{g=1}{\prod }}\eta_{hg}, \end{array}$$

where *F*(·) is a specific linking function and β_*jc*0_, β_*jcg*_, and $\beta_{jcgg^{\prime }}$ are the baseline, main, and interaction effects for Category *c*, respectively. Some widely used linking functions include the identity, logit, and log links. The conversion from *P*_*c*_(**η**_*jh*_) to different item effects β_*jc*._ can be facilitated with a design matrix as discussed later. Note that model-data fit for different linking functions in saturated forms is theoretically identical since no estimation is involved. For dichotomous responses, the formulation is equivalent to the G-DINA model with the identity link or LCDM with the logit link functions.

### Model estimation

The above GPDM, in the saturated form with the identity linking function, can be estimated with the MML method and implemented using an EM algorithm. A large number of structural parameters can be involved as the number of attributes increases. Although it is possible to simplify the joint distribution of the attributes with specific constraints on the structural relationships (e.g., higher-order or hierarchical structures) in future studies, this research only considers the general or unconstrained structure. In general, there are (*L*-1) independent structural parameters *p*(**α**_*l*_), which is the prior probability of the attribute vector α_*l*_ with $\sum_{l=1}^{L}p\left(\alpha_{l}\right)=1$. Denote *X*_*ijc*_ = 1 if *X*_*ij*_ = *c* and zero otherwise. The conditional likelihood of the response data **X**_*i*_ for examinee *i* given α_*l*_ is

(3) $$\begin{array}{r} L\left({\text{X}}_{i}|\alpha_{l}\right)=\prod_{j=1}^{J}\prod_{c=0}^{C_{j}-1}P_{c}{\left(\eta_{jh}\right)}^{X_{ijc}} \end{array}$$

where the attribute vector **α**_*l*_ is reduced to the reduced attribute vector **η**_*jh*_ for item *j*. The marginalized likelihood of the data is

(4) $$\begin{array}{r} L\left(\text{X}\right)=\prod_{i=1}^{N}L\left({\text{X}}_{i}\right)=\prod_{i=1}^{N}\sum_{l=1}^{L}L\left({\text{X}}_{i}|\alpha_{l}\right)p\left(\alpha_{l}\right), \end{array}$$

where *N* is the sample size. The MML estimation of the conditional probabilities ${\hat{P}}_{c}\left(\eta_{jh}\right)$ can be implemented with an EM algorithm. The standard error (SE) of the estimate, $\mathrm{SE}\left[{\hat{P}}_{c}\left(\eta_{jh}\right)\right]$, can be approximated with the information matrix given by the second derivative of the log-marginalized likelihood with respect to *P*_*c*_(**η**_*jh*_) and $P_{c^{\prime }}\left(\eta_{jh^{\prime }}\right)$. Details of an algorithm for the estimates ${\hat{P}}_{c}\left(\eta_{jh}\right)$ and corresponding SEs can be found in the Appendix in Supplementary Material.

During each iteration of the estimation, the monotonicity assumption can be imposed top-down as a constraint with the help of (1). For an item with four categories for instance, one can first impose the constraint on the top category with $P_{3}^{*}\left(\eta_{jh}\right)=P_{3}\left(\eta_{jh}\right)$, and then on the second top category with $P_{2}^{*}\left(\eta_{jh}\right)=P_{2}\left(\eta_{jh}\right)+P_{3}^{*}\left(\eta_{jh}\right)$, till the second lowest category with $P_{1}^{*}\left(\eta_{jh}\right)=P_{1}\left(\eta_{jh}\right)+P_{2}^{*}\left(\eta_{jh}\right)$. For each category, one can choose between the upward or downward approaches to implement the constraint, although the upward one would be generally preferred as in the case for dichotomous response (Hong et al., 2016). Alternatively, one can choose to check the monotonicity assumption *post hoc* (i.e., after the estimation), which could help to inform item development.

### Different saturated and reduced forms

The GPDM is a saturated model, with equivalent saturated forms, and subsumes a variety of reduced CDMs for polytomous and dichotomous responses. For different saturated forms and some reduced forms, the estimates ${\hat{P}}_{c}\left(\eta_{jh}\right)$ can be linearly transformed to item parameters (i.e., item effects or β_*jc*._) without requiring additional estimation. Corresponding linking functions and design matrices somewhat similar to those in the G-DINA model (de la Torre, 2011) can be used. The design matrix for item *j*, **D**_*j*_, is a *H*_*j*_ × *V* matrix, where *V* is the number of item parameters of the targeted form. For other reduced forms however, the item parameters might involve a second step of estimation from ${\hat{P}}_{c}\left(\eta_{jh}\right)$, which is beyond the scope of this research.

For saturated forms, the design matrix is a square matrix with element

(5) $$d_{hh^{\prime }}=\{\begin{array}{c} 1\\ 0 \end{array}\begin{array}{c} \eta_{jh}\eta_{jh^{\prime }}^{T}=G_{j}\\ otherwise \end{array},$$

where *h* and $h^{\prime }=1,\ldots ,H_{j}$. The item parameters can be obtained by finding the least-squares estimate

(6) $$\begin{array}{r} {\hat{\beta }}_{jc}={\left(D_{j}^{T}{\text{D}}_{j}\right)}^{-1}{\text{D}}_{j}^{T}F\left({\hat{\text{P}}}_{jc}\right), \end{array}$$

where F(.) is the linking function (e.g., identity, logit, or log) and $P_{jc}={\left\{P_{c}(\eta_{jh})\right\}}_{jc}$ includes all independent *P*_*c*_(**η**_*jh*_) for item *j*. The standard errors can be approximated via the multivariate delta method (Lehmann and Casella, 1998). Denote $f\left(\cdot \right)\equiv {\left({\text{D}}_{j}^{T}{\text{D}}_{j}\right)}^{-1}{\text{D}}_{j}^{T}F\left(\cdot \right)$ and ∇*f*(·) as the gradient of *f* (.). We have

(7) $$\begin{array}{r} \mathrm{SE}{\left({\hat{\beta }}_{jc}\right)}^{2}=\mathrm{Var}\left[f\left({\hat{\text{P}}}_{jc}\right)\right]\approx \nabla f{\left({\hat{\text{P}}}_{jc}\right)}^{T}\mathrm{Var}\left({\hat{\text{P}}}_{jc}\right)\nabla f\left({\hat{\text{P}}}_{jc}\right), \end{array}$$

where $\mathrm{Var}\left({\hat{\text{P}}}_{jc}\right)$ is the negative of the inverse of the information matrix for ${\hat{\text{P}}}_{jc}$.

By constraining the item parameters, one can get different reduced CDMs for polytomous responses. The DINA model for polytomous responses (PDINA) can be expressed as

(8) $$\begin{array}{r} P_{c}^{}\left(\eta_{jh}\right)=\beta_{jc0}+\beta_{jc12\ldots G_{j}}\overset{G_{j}}{\underset{g=1}{\prod }}\eta_{hg}, \end{array}$$

with 2(*C*_*j*_ – 1) parameters per item. The conjunctive attribute relationship in the DINA model is extended to every above-zero category in the PDINA model. Consistent with terminology in previous literature, we can set *g*_*jc*_ = β_*jc*0_ and *s*_*jc*_ = (1 − β_*jc*0_ − β_*jc*12…_*G*__*j*__) as the guessing and slip parameter for Category *c*, respectively. Similarly, one can obtain the DINO model for polytomous responses (PDINO) by adopting the identity link with

(9) $$\begin{array}{r} P_{c}^{}\left(\eta_{jh}\right)=\beta_{jc0}+\beta_{jcg}\eta_{hg}, \end{array}$$

where

(10) $$\begin{array}{r} \beta_{jcg}=\beta_{jcg^{\prime }g^{\prime \prime }}=\ldots ={\left(-1\right)}^{G_{j}+1}\beta_{jc12\ldots G_{j}}, \end{array}$$

for *g* = 1, …, *G*_*j*_, $g^{\prime }=1,\ldots ,G_{j}-1$, and $g^{\prime \prime }>g^{\prime },\ldots ,G_{j}$. PDINO also has 2(*C*_*j*_ – 1) parameters per item, and the compensatory attribute relationship in the DINO model is extended to every above-zero categories in the PDINO model. For this model, *g*_*jc*_ = β_*jc*0_ and *s*_*jc*_ = (1 − β_*jc*0_ − β_*jcg*_) is the guessing and slip parameter respectively, for Category *c*. The item parameters of both the PDINA and PDINO models can be linearly transformed from the ${\hat{\text{P}}}_{jc}$ using the design matrices, which are

(11) $$\begin{array}{r} {\text{D}}_{j\left[H_{j}\times 2\right]}=\left(\begin{array}{cc} 1 & 0\\ 1 & 0\\ \vdots & \vdots \\ 1 & 0\\ 1 & 1 \end{array}\right) \end{array}$$

and

(12) $$\begin{array}{r} {\text{D}}_{j\left[H_{j}\times 2\right]}=\left(\begin{array}{cc} 1 & 0\\ 1 & 1\\ \vdots & \vdots \\ 1 & 1\\ 1 & 1 \end{array}\right) \end{array}$$

for the PDINA and PDINO models, respectively. Moreover, to account for the relative size of the latent class, the item parameters of the above models should be weighted by the class size, as:

(13) $$\begin{array}{r} {\hat{\beta }}_{jc}={\left({\text{D}}_{j}^{T}{\text{W}}_{j}{\text{D}}_{j}\right)}^{-1}{\text{D}}_{j}^{T}{\text{W}}_{j}{\hat{\text{P}}}_{jc} \end{array}$$

where **W**_*j*_ = diag{*N*_*jh*_} is a diagonal matrix, with $N_{jh}=\sum_{i=1}^{N}p\left(\eta_{jh}|{\text{X}}_{i}\right)$ as the expected number of examinees in latent group *h* of item *j* (i.e., with the reduced attribute vector η_*jh*_). The standard errors of these estimates can also be computed as given in (7), except that $f\left({\hat{\text{P}}}_{jc}\right)$ is defined as ${\left({\text{D}}_{j}^{T}{\text{W}}_{j}{\text{D}}_{j}\right)}^{-1}{\text{D}}_{j}^{T}{\text{W}}_{j}{\hat{\text{P}}}_{jc}$. Similar to the cases for dichotomous responses, a linear transformation of the saturated models to the reduced models is equivalent to a direct estimation of the reduced models using the same MML method (de la Torre and Chen, 2011).

However, not all reduced forms can be obtained through linear transformation. By dropping all interaction terms in (2), one can obtain different additive forms of CDMs for polytomous responses, as

(14) $$\begin{array}{r} F\left[P_{c}^{}\left(\eta_{jh}\right)\right]=\beta_{jc0}+\overset{G_{j}}{\underset{g=1}{\sum }}\beta_{jcg}\eta_{hg} \end{array}$$

The formulation with the identity, logit, and log linking function corresponds to the A-CDM for polytomous response (PA-CDM), LLM for polytomous responses (PLLM), and R-RUM for polytomous responses (PR-RUM), respectively. The additive forms have a common feature that there are (*C*_*j*_ − 1)(*G*_*j*_ + 1) parameters or effects per item. Although the additive forms are subsumed under the GPDM, linear relationship between the (*C*_*j*_ − 1)(*G*_*j*_ + 1) item parameters and (*C*_*j*_ − 1)*H*_*j*_ ${\hat{P}}_{c}\left(\eta_{jh}\right)$ estimates cannot be established, except for the case of a single attribute. Additional estimation analyses are required to obtain the item parameters of these reduced forms, which is not covered in this research but could be a topic for future studies.

### Model assessment and adjustment

Model assessment and adjustment for dichotomous responses can be extended to polytomous responses. This research builds on the simulation- and residual-based method (Chen et al., 2013) and applies the correlation of item pairs for such purpose. Specifically, after obtaining the estimates *P*_*c*_(**η**_*jh*_) and *p*(**α**_*l*_|**X**), one can simulate a large number of item responses by sampling from the multinomial distribution of the conditional probabilities and joint distribution of the attributes. Let *X*_*j*_ and ${\tilde{X}}_{j}$ denote the observed and predicted response vector for item *j*, respectively. The residual between the observed and predicted Fisher-transformation of correlation of item pairs (referred to as *r*) is

(15) $$\begin{array}{r} r_{jj^{\prime }}=|Z\left[\rho \left({\text{X}}_{j},{\text{X}}_{j^{\prime }}\right)\right]-Z\left[\rho \left({\tilde{\text{X}}}_{j},{\tilde{\text{X}}}_{j^{\prime }}\right)\right]|, \end{array}$$

where *Z*[ρ(·)] is the Fisher transformation of Pearson's correlation. The approximate SE can be computed as:

(16) $$\begin{array}{r} SE\left(r_{jj^{\prime }}\right)={\left[N-3\right]}^{1/2}. \end{array}$$

With the SEs, the *z*-score of *r* can be computed. The expected response patterns can be simulated stably with a large sample size, although randomness is inevitable due to simulation. For an assessment with *J* items, (*J* – 1) and *J*(*J* – 1)/2 item pairs of z-scores need to be evaluated at the item and test levels, respectively.

One can examine the significance of the maximum z-score for model misfit at the test level, with the Bonferroni correction to control for the Type I errors (Chen et al., 2013). In case of misfit, we can use the root mean square (RMS) of the z-scores at the item level to identify possibly misspecified items, as

(17) $$\begin{array}{r} sr_{j}={\left[\sum_{j^{\prime }=1}^{J}{\left(r_{jj^{\prime }}/SE\left(r_{jj^{\prime }}\right)\right)}^{2}/\left(J-1\right)\right]}^{1/2} \end{array}$$

The item with the maximum value *sr*_*j*_ (called the hit item) is most likely misspecified and should be considered for adjustment. The test-level RMS of the z-scores is

(18) $$\begin{array}{r} sr={\left[2\sum_{j=1}^{J}\sum_{j^{\prime }=1}^{j-1}{\left(r_{jj^{\prime }}/SE\left(r_{jj^{\prime }}\right)\right)}^{2}/J\left(J-1\right)\right]}^{1/2} \end{array}$$

*sr* should be less sensitive to simulation randomness due to the accumulation of all residuals z-scores, and its reduction can aid in item adjustment. Findings for dichotomous responses (Chen, 2017) can be applied to polytomous responses. Misspecified items can be evaluated as the hit item and adjusted sequentially when Q-matrix misspecification occurs at the item level. Adjustments can proceed based on the maximum reduction of the *sr* statistics. Attribute-level misspecification is of concern when adjustment to individual items tends to be useless.

## Simulation study

### Design

A simulation study was conducted to investigate if the GPDM can perform as expected. The true CDM was the PA-CDM with the identity link, and *C*_*j*_ = *C* = 3 for all items. The cumulative probabilities of the highest category, $P_{2}^{*}\left(0\right)$ and $P_{2}^{*}\left(1\right)$, were randomly generated from *Unif* (0.0, 0.3) and *Unif* (0.7, 1.0), respectively, with those of the middle category [i.e., $P_{1}^{*}\left(0\right)$ and $P_{1}^{*}\left(1\right)$] fixed as half of the highest category. It means that the conditional probabilities of both categories were equally contributed [i.e., *P*_1_(**0**) = *P*_2_(**0**) and *P*_1_(**1**) = *P*_2_(**1**)]. All main effects (i.e.,β_*jk*_) were set to be equal so that each required attribute contributed equally to *P*_*c*_(**η**_*jh*_). The number of attributes *K* was fixed to five and the Q-matrix consisted of one- to three-attribute items, with each attribute specified the same number of times (Table 1). The number of items was set as *J* = 20 and 40, and two sample sizes were considered as *N* = 500 and 1,000. The Q-matrix for *J* = 40 is just twice the Q-matrix for *J* = 20.

**Table 1:** Q-matrix for *J* = 20.

	**Attribute**						**Attribute**				
**Item**	**α_1_**	**α_2_**	**α_3_**	**α_4_**	**α_5_**	**Item**	**α_1_**	**α_2_**	**α_3_**	**α_4_**	**α_5_**
1	1	0	0	0	0	11	1	1	1	0	0
2	0	1	0	0	0	12	1	1	0	0	1
3	0	0	1	0	0	13	1	0	0	1	1
4	0	0	0	1	0	14	0	1	1	1	0
5	0	0	0	0	1	15	0	0	1	1	1
6	1	1	0	0	0	16	1	0	1	0	0
7	1	0	0	0	1	17	1	0	0	1	0
8	0	1	1	0	0	18	0	1	0	1	0
9	0	0	1	1	0	19	0	1	0	0	1
10	0	0	0	1	1	20	0	0	1	0	1

*The Q-matrix for J = 40 was duplicated*.

The multivariate normal threshold method (Chiu et al., 2009) was used to simulate the joint distribution of the attributes. One can assume a multivariate normal distribution, *MVN*(**0**, ***Σ***), of *K* continuous latent variables underlying the *K* discrete attributes, with all variances and covariance in ***Σ*** equal to 1.0 and *R*, respectively. *R* is the correlations of the latent variables and was set as 0.5. In addition to the GPDM, the G-DINA model was also fitted to allow for comparisons. For the G-DINA model, the polytomous responses were converted to dichotomous by replacing responses in the highest category with 1 and those in other categories with 0. Each simulation cell was replicated 500 times, and the estimation code was written in Ox (Doornik, 2003)^1^.

### Results

Table 2 gives the mean estimates and related standard deviation (SD) of the classification accuracy for individual attributes [CA(α_*k*_)] and for the attribute vector [CA(α_*l*_)]. For both individual attributes and the attribute vector, the accuracy improved with the larger sample size or number of items. The number of items was more influential, especially on the attribute vector. Comparing the two models, the GPDM always produced more accurate classifications than the G-DINA model did, and the improvement was more dramatic for the attribute vector (about double or more) than individual attributes. The findings simply reflected the loss of diagnostic information by treating the polytomous responses as dichotomous under the settings used in this study. The classifications were also more stable (i.e., smaller SD) with either larger sample size or larger number of items for the GPDM, with the latter being more influential. For the G-DINA model, the classifications of the attribute vector were found to be more stable with a smaller number of items, or compared with those for the GPDM. Taking into account the CA(α_*l*_) values however, the stability suggested that the G-DINA-based accuracy was consistently low under the situation.

**Table 2:** Classification accuracy and related SD.

		**GPDM**				**G-DINA**			
***J***	***N***	**CA(α*_*l*_*)**	**CA(α*_*k*_*)**	**SD(α*_*l*_*)**	**SD(α*_*k*_*)**	**CA(α*_*l*_*)**	**CA(α*_*k*_*)**	**SD(α*_*l*_*)**	**SD(α*_*k*_*)**
20	500	0.609	0.901	0.055	0.035	0.257	0.729	0.031	0.041
	1000	0.643	0.911	0.051	0.030	0.281	0.747	0.030	0.036
40	500	0.848	0.966	0.033	0.017	0.381	0.811	0.044	0.037
	1000	0.865	0.970	0.029	0.014	0.440	0.837	0.033	0.026

*For α_k_, the values are averaged across all individual attributes*.

Although one cannot investigate the recovery of all item parameters as the true and fitted models were different, some item estimates can be evaluated. Specifically, the true and estimate *P*_*c*_(**0**) and *P*_*c*_(**1**) should be approximately identical with the GPDM. Table 3 gives the mean bias and root mean square error (RMSE) of the estimates of the conditional probabilities. As shown, both statistics improved with the larger sample size or number of items, with the former being slightly more influential. The item parameters were recovered equally well across the two response categories, reflecting the design of equal contribution of both categories.

**Table 3:** Recovery of item estimates with the GPDM.

		**Mean bias**				**RMSE**			
***J***	***N***	***P*_1_(0)**	***P*_1_(1)**	***P*_2_(0)**	***P*_2_(1)**	***P*_1_(0)**	***P*_1_(1)**	***P*_2_(0)**	***P*_2_(1)**
20	500	0.008	0.008	−0.007	−0.008	0.027	0.027	0.044	0.044
	1000	0.005	0.005	−0.005	−0.005	0.018	0.019	0.031	0.031
40	500	0.003	0.004	−0.005	−0.004	0.022	0.022	0.039	0.039
	1000	0.002	0.003	−0.002	−0.003	0.015	0.015	0.028	0.028

*All values are averaged across items*.

## Real-data illustration

In this section real data were used to illustrate the application of the GPDM in practice and how different results one might get whether polytomous items were dichotomized or not. The PISA 2000 reading assessment (OECD, 1999, 2006a) with the released items (OECD, 2006b) was used, which contained both polytomous and dichotomous items. The data were retrofitted by some researchers with a new set of attributes (Chen and Chen, 2015), but only part of the released items were dichotomously fitted with the G-DINA model. With the same set of attributes and the help of same content experts, a subset of the released items was used to construct a Q-matrix (Table 4) for illustration purpose. The final data set consisted of responses of 1,039 English examinees to 20 items in specific test booklet, five out of which were polytomous. Both the GPDM and G-DINA model were fitted. For the G-DINA model, the polytomous items were again dichotomized by setting the highest level to 1 and all other levels to 0.

**Table 4:** Items and Q-matrix for the PISA data.

**No**.	**Code**	***C***	**α_1_**	**α_2_**	**α_3_**	**α_4_**	**α_5_**	**No**.	**Code**	***C***	**α_1_**	**α_2_**	**α_3_**	**α_4_**	**α_5_**
1	R040Q02	2	1	0	1	0	0	11	R088Q04T	3	1	0	1	0	0
2	R040Q03A	2	1	0	1	1	0	12	R088Q05T	2	0	1	1	1	0
3	R040Q04	2	0	1	1	1	0	13	R088Q07	2	0	1	0	0	1
4	R040Q06	2	1	0	1	0	0	14	R216Q01	2	0	1	0	0	0
5	R077Q03	3	0	1	0	1	1	15	R216Q02	2	1	0	0	0	1
6	R077Q04	2	1	1	1	0	0	16	R216Q03T	2	0	1	1	0	0
7	R077Q05	3	0	1	1	1	0	17	R216Q04	2	0	1	1	0	0
8	R077Q06	2	0	1	0	0	1	18	R216Q06	2	0	1	0	1	0
9	R088Q01	2	0	1	1	0	0	19	R236Q01	2	1	0	1	0	0
10	R088Q03	3	1	0	1	0	0	20	R236Q02	3	0	0	1	1	0

*C, number of categories; α_1_, retrieving information; α_2_, forming a broad general understanding; α_3_, developing an interpretation; α_4_, reflecting on and evaluating the content of a text; α_5_, reflecting on and evaluating the form of a text*.

Table 5 presents the results of model fitting and attribute estimation. For both models, the maximum z-score of *r* were well below the critical value at α = 0.1 significance level, suggesting that no misfit could be found for either model. However, there was slight to moderate difference in classifications across attributes between the two models, as shown by the attribute prevalence and classification consistency based on Cohen's kappa (to adjust for chance of consistency). The difference was most salient for Attribute 4, suggesting that the loss of information was most severe for this attribute if polytomous responses were dichotomized. As a result, only 68% of examinees were identically classified (i.e., same attribute vector) across the two models.

**Table 5:** Fitting and attribute estimation for the PISA data.

			**Attribute prevalence**					**Cohen's kappa**					**CC**
**Model**	***z_*c*_***	***mzr***	***p*(α_1_)**	***p*(α_2_)**	***p*(α_3_)**	***p*(α_4_)**	***p*(α_5_)**	**α_1_**	**α_2_**	**α_3_**	**α_4_**	**α_5_**	**α*_*l*_***
GPDM	3.47	2.54	0.66	0.68	0.53	0.65	0.49	0.89	0.94	0.89	0.49	0.92	0.68
G-DINA	3.47	2.88	0.63	0.69	0.56	0.46	0.51	–	–	–	–	-	-

*z_c_, critical z-score at 10% significance level (the Bonferroni correction); mzr, maximum z-score of r; CC, classification consistency*.

Table 6 presents the estimates of the polytomous items using the GPDM. Except for the last item, number 20, at least some of the conditional probabilities for the middle response category [i.e.,*P*_1_(**η**_*jh*_)] were not trivial. Among these polytomous items, examinees were most likely to get partial credit for Item 10 and 11. For both items, the conditional probabilities *P*_1_(10) or *P*_1_(01) > *P*_1_(11) or *P*_1_(00), whereas the cumulative probabilities $P_{1}^{*}\left(11\right)>P_{1}^{*}$(10) or $P_{1}^{*}\left(01\right)$. In reflection, this implied that the middle category (i.e., partial credit) was specifically oriented toward examinees mastering some but not all required attributes, which can be regarded as a useful way to design the item category. For Item 5 and 7, there was little chance to get partial credit for some reduced attribute patterns (e.g., 000). The small values of all *P*_1_(**η**_*jh*_) for Item 20 suggested that the middle category was unnecessary. Finally, the monotonicity assumption was not imposed, and one can find that there were some mild violations in Item 5 [e.g., $P_{2}^{*}\left(001\right)>P_{2}^{*}\left(101\right)$ or $P_{2}^{*}\left(011\right)$] and Item 7 [e.g., $P_{1}^{*}\left(001\right)>P_{1}^{*}\left(110\right)$ or $P_{1}^{*}\left(011\right)$], which might be useful information to adjust the items.

**Table 6:** Estimates of conditional probabilities for polytomous items.

		***p***_*****c*****_(η_*****jh*****_)								**Required attributes**				

		**(00)**	**(10)**	**(01)**	**(11)**									
**Item**	***c***	**(000)**	**(100)**	**(010)**	**(001)**	**(110)**	**(101)**	**(011)**	**(111)**	α_1_	α_2_	α_3_	α_4_	α_5_
5	0	0.90	0.38	0.37	0.26	0.20	0.54	0.50	0.05	0	1	0	1	1
	1	0.02	0.05	0.29	0.10	0.20	0.21	0.25	0.04					
	2	0.08	0.57	0.34	0.64	0.60	0.24	0.25	0.90					
7	0	0.92	0.47	0.58	0.29	0.37	0.34	0.50	0.23	0	1	1	1	0
	1	0.06	0.33	0.20	0.40	0.46	0.23	0.25	0.18					
	2	0.02	0.20	0.21	0.32	0.17	0.43	0.25	0.60					
10	0	0.59	0.21	0.15	0.09					1	0	1	0	0
	1	0.38	0.62	0.61	0.28									
	2	0.02	0.16	0.24	0.63									
11	0	0.72	0.29	0.47	0.14					1	0	1	0	0
	1	0.27	0.67	0.48	0.48									
	2	0.02	0.04	0.05	0.38									
20	0	0.99	0.96	0.92	0.52					0	0	1	1	0
	1	0.01	0.03	0.01	0.06									
	2	0.01	0.01	0.07	0.42									

*Numbers with parentheses, e.g., (00) or (000), represent different attribute vectors, with the first row for two-attribute items and the second row for three-attribute items*.

Table 7 compares the model-data fits between the saturated GPDM and the reduced PDINA or PDINO models. Both reduced models were worse than the saturated model based on any of the relative fit indices. Furthermore, both the PDINA and PDINO models were rejected at α = 0.01 significance level based on the maximum z-score, which suggests that highly constrained relationships are not appropriate. It would be interesting to see how less-restricted reduced models (e.g., linear models such as the PLLM or PA-CDM) will perform under similar context in future studies, when an estimation algorithm is available for those models.

**Table 7:** Model-data fit comparisons between the saturated and reduced models.

**Model**	***mzr***	***sr***	**NP**	**−2LL**	**AIC**	**BIC**
GPDM	2.54	0.89	161	25,179	25,501	26,297
PDINA	9.22	4.45	81	26,488	26,650	27,051
PDINO	10.36	4.20	81	26,568	26,730	27,130

*Mzr, maximum z-score; the critical z-score was 4.04 at 0.01 significance level; sr, test-level RMS of the z-scores; NP, number of parameters; −2LL = −2 × log-likelihood; AIC, Akaike's information criterion (Akaike, 1974); BIC, Bayesian information criterion (Schwarzer, 1976)*.

## Discussion

Although considerable developments have been added to the CDM literature recently, most have been conducted for dichotomous responses only. Without a comprehensive modeling framework for polytomous responses, the advance of CDMs cannot be fully appreciated by applied researchers and practitioners across a wide range of areas. This research proposes a general cognitive diagnosis model for polytomous responses, which split the polytomous items indirectly based on the difference-between-category approach. The GPDM can be regarded as a natural extension of the G-DINA model, and the MML estimation is implemented using an EM algorithm. Under the GPDM framework, different saturated forms, and some reduced forms, can be transformed linearly. Model assessment and adjustment under the context of polytomous responses can proceed with the simulation- and residual-based method using the correlation of item pairs. The simulation study demonstrated the effectiveness of the model, especially when compared to dichotomizing polytomous responses with the G-DINA model. The improvement of classification accuracy of the attribute vector was dramatic with the use of the GPDM. Note that the number of items was more influential than the sample size on classification accuracy. This seems to be a good news for practitioners as increasing the number of items administered is usually more controllable in practice. Some item parameters of reduced CDMs for polytomous responses can be also recovered well with the GPDM. The real-data example further illustrated how the GPDM can make a difference in practice by providing additional diagnostic information and contributing to item development.

Although the GPDM appears to be promising, some issues should be addressed in future research for the framework to be more comprehensive and versatile. First, various reduced forms are subsumed under the framework and it would be useful to study some reduced CDMs in more details. As the number of response categories increases, the GPDM gets more complex, with a large number of parameters, where reduced models have fewer parameters and hence require smaller sample sizes for accurate estimation. Reduced models also have more straightforward interpretation of the attribute-item relationships when the models fit the data. In this regard, it would be helpful to investigate how various reduced models can effectively be estimated and compared within the general framework. Second, it would be valuable to assess whether Q-matrix validation procedures under the dichotomous context can be extended to polytomous responses, as Q-matrix plays a critical role in CDM-based measurement. It is also useful extend efficient and exhaustive search algorithm similar to the general method based on the discrimination index (de la Torre and Chiu, 2015) to polytomous responses. Third, the GPDM represents a specific approach to modeling polytomous responses. It would be interesting to compare other approaches of modeling to see how they could differ. Moreover, it is desirable to develop CDMs that can accommodate various response formats already covered under the IRT context (e.g., nominal, forced choice, graded unfolding), which can offer applied researchers a new perspective to re-conceptualize their research. Finally, the current treatment of the issue tended to be statistically oriented and compact. It would be desirable to have a didactic companion expanding and illustrating the modeling processes and procedures of model assessments and adjustments for practitioners. This would be especially useful when effective estimation methods for various reduced models are available.

## Author contributions

JC was responsible for most parts of the research, while JdlT helped with the introduction and discussion parts.

### Conflict of interest statement

The authors declare that the research was conducted in the absence of any commercial or financial relationships that could be construed as a potential conflict of interest.

## Footnotes

^1^The updated estimation code in Ox is available from the first author.
