# Supplementary material for: Introducing the General Polytomous Diagnosis Modeling Framework
Source: Front Psychol. 2018 Aug 22;9:1474. doi: 10.3389/fpsyg.2018.01474 (PMC6113892; doi:10.3389/fpsyg.2018.01474)
Supplement: Supplementary file 1 [file Presentation_1.pdf]

Appendix

A MML-EM Algorithm for the GPDM

A1. Parameter Estimation

The log of the marginalized likelihood of the data is

$$l(\mathbf{X}) = \log \prod_{i=1}^{N} L(\mathbf{X}_i) = \sum_{i=1}^{N} \log L(\mathbf{X}_i)\,. \quad \text{(A1)}$$

Let $p_{jhc} = P_c(\boldsymbol{\eta}_{jh})$, the derivative with respect to p_{jhc} is

$$\frac{\partial l(\mathbf{X})}{\partial p_{jhc}} = \sum_{i=1}^{N} \frac{1}{L(\mathbf{X}_i)} \frac{\partial L(\mathbf{X}_i)}{\partial p_{jhc}} = \sum_{i=1}^{N} \frac{1}{L(\mathbf{X}_i)} \sum_{\{l:\boldsymbol{\alpha}_l = \boldsymbol{\eta}_{jh}\}} p(\boldsymbol{\alpha}_l) \frac{\partial L(\mathbf{X}_i \mid \boldsymbol{\alpha}_l)}{\partial p_{jhc}}\,, \quad \text{(A2)}$$

where only attribute patterns $\{l\text{: } \boldsymbol{\alpha}_l = \boldsymbol{\eta}_{jh}\}$ is relevant. Meanwhile, we have

$$\frac{\partial L(\mathbf{X}_i \mid \boldsymbol{\alpha}_l)}{\partial p_{jhc}} = \prod_{j \neq j'} \prod_{c'=0}^{C_j - 1} (p_{j'hc'})^{X_{ij'c'}} \frac{\partial \prod_{c'=0}^{C_j - 1} (p_{jhc'})^{X_{ijc'}}}{\partial p_{jhc}}\,. \quad \text{(A3)}$$

One can set $p_{jh0} = 1 - \sum_{c'=1}^{C_j - 1} p_{jhc'}$. Then $p_{jh1}, p_{jh2}, \cdots, p_{jh(C_j - 1)}$ are independent, with

$\frac{\partial p_{jh0}}{\partial p_{jhc}} = -1$ for $c > 0$. So, the derivative in the right-hand side of (A3) is

$$\begin{aligned}
&\frac{\partial \prod_{c'=0}^{C_j - 1} (p_{jhc'})^{X_{ijc'}}}{\partial p_{jhc}} \\
&= \frac{\partial \left[(p_{jh0})^{X_{ij0}} \right]}{\partial p_{jhc}} \prod_{c'=1}^{C_j - 1} (p_{jhc'})^{X_{ijc'}} + \frac{\partial \left[\prod_{c'=1}^{C_j - 1} (p_{jhc'})^{X_{ijc'}} \right]}{\partial p_{jhc}} (p_{jh0})^{X_{ij0}} \\
&= \prod_{c'=0}^{C_j - 1} (p_{jhc'})^{X_{ijc'}} \left(\frac{X_{ijc}}{p_{jhc}} - \frac{X_{ij0}}{p_{jh0}} \right). \quad \text{(A4)}
\end{aligned}$$

Replacing (A4) with the derivative in the right-hand side of (A3), we have

$$\frac{\partial L(\mathbf{X}_i \mid \boldsymbol{\alpha}_l)}{\partial p_{jhc}} = L(\mathbf{X}_i \mid \boldsymbol{\alpha}_l) \left(\frac{X_{ijc}}{p_{jhc}} - \frac{X_{ij0}}{p_{jh0}} \right). \quad \text{(A5)}$$

And substituting (A5) into (A2) give us

$$\frac{\partial l(\mathbf{X})}{\partial p_{jhc}} = \sum_{i=1}^{N} \sum_{\{l:\boldsymbol{\alpha}_l = \boldsymbol{\eta}_{jh}\}} \frac{p(\boldsymbol{\alpha}_l) L(\mathbf{X}_i \mid \boldsymbol{\alpha}_l)}{L(\mathbf{X}_i)} \left(\frac{X_{ijc}}{p_{jhc}} - \frac{X_{ij0}}{p_{jh0}} \right)$$
$$= \sum_{i=1}^{N} \sum_{\{l:\boldsymbol{\alpha}_l = \boldsymbol{\eta}_{jh}\}} p(\boldsymbol{\alpha}_l \mid \mathbf{X}_i) \left(\frac{X_{ijc}}{p_{jhc}} - \frac{X_{ij0}}{p_{jh0}} \right), \qquad \text{(A6)}$$

where $p(\boldsymbol{\alpha}_l \mid \mathbf{X}_i)$ is the posterior probability of examinee i for attribute vector $\boldsymbol{\alpha}_l$. $p(\boldsymbol{\alpha}_l \mid \mathbf{X}_i)$ can be further reduced to $p(\boldsymbol{\eta}_{jh} \mid \mathbf{X}_i)$, the posterior probability of examinee i for $\boldsymbol{\eta}_{jh}$ with the q-vector $\mathbf{q}_j$, and (A6) can be written as

$$\frac{\partial l(\mathbf{X})}{\partial p_{jhc}} = \sum_{i=1}^{N} \sum_{\{l:\boldsymbol{\alpha}_l = \boldsymbol{\eta}_{jh}\}} \left(\frac{p(\boldsymbol{\eta}_{jh} \mid \mathbf{X}_i) X_{ijc}}{p_{jhc}} - \frac{p(\boldsymbol{\eta}_{jh} \mid \mathbf{X}_i) X_{ij0}}{p_{jh0}} \right)$$
$$= \frac{\sum_{i=1}^{N} p(\boldsymbol{\eta}_{jh} \mid \mathbf{X}_i) X_{ijc}}{p_{jhc}} - \frac{\sum_{i=1}^{N} p(\boldsymbol{\eta}_{jh} \mid \mathbf{X}_i) X_{ij0}}{p_{jh0}}. \qquad \text{(A7)}$$

If we denotes $N_{jh} = \sum_{i=1}^{N} p(\boldsymbol{\eta}_{jh} \mid \mathbf{X}_i)$ as the expected number of examinees with the reduced attribute vector $\boldsymbol{\eta}_{jh}$, and $R_{jhc} = \sum_{i=1}^{N} p(\boldsymbol{\eta}_{jh} \mid \mathbf{X}_i) X_{ijc}$ as the expected number of examinees with $\boldsymbol{\eta}_{jh}$ responding c to item j, we will obtain

$$\frac{\partial l(\mathbf{X})}{\partial p_{jhc}} = \frac{R_{jhc}}{p_{jhc}} - \frac{R_{jh0}}{p_{jh0}}. \qquad \text{(A8)}$$

The MML estimation with respect to p_{jhc} is equivalent to solve for $\frac{\partial l(\mathbf{X})}{\partial p_{jhc}} = 0$, which gives

$$\frac{R_{jhc}}{p_{jhc}} = \frac{R_{jh0}}{p_{jh0}}, \qquad \text{(A9)}$$

for $c = 1, \ldots, C_j - 1$. Alternative, (A9) can be re-expressed as

$$\frac{R_{jh0}}{p_{jh0}} = \frac{R_{jh1}}{p_{jh1}} = \cdots \frac{R_{jhc}}{p_{jhc}} = \cdots \frac{R_{jhC_j-1}}{p_{jhC_j-1}} = \frac{\sum_{c'=0}^{C_j-1} R_{jhc'}}{\sum_{c'=0}^{C_j-1} p_{jhc'}} = N_{jh}. \qquad \text{(A10)}$$

Therefore,

$$\hat{p}_{jhc} = \hat{P}_c\,(\boldsymbol{\eta}_{jh}) = R_{jhc} / N_{jh}\,, \tag{A11}$$

or equivalently,

$$\hat{P}_c^*(\boldsymbol{\eta}_{jh}) = \sum\nolimits_{c'=c}^{C_j-1} R_{jhc'} / N_{jh}\,. \tag{A12}$$

During the estimation process, the item estimates and posterior probabilities can be iteratively updated using the empirical Bayes method (Deely & Lindley, 1981). Specifically, the posterior probability of examine i for $\boldsymbol{\alpha}_l$ at the tth iterative estimation process is updated as:

$$p(\boldsymbol{\alpha}_l \mid \mathbf{X}_i)_t = \frac{L(\mathbf{X}_i \mid \boldsymbol{\alpha}_l)\,p(\boldsymbol{\alpha}_l \mid \mathbf{X}_i)_{t-1}}{\sum\nolimits_{l'=1}^{L} L(\mathbf{X}_i \mid \boldsymbol{\alpha}_{l'})\,p(\boldsymbol{\alpha}_{l'} \mid \mathbf{X}_i)_{t-1}}\,, \tag{A13}$$

where $p(\boldsymbol{\alpha}_l \mid \mathbf{X}_i)_{t=0} = p(\boldsymbol{\alpha}_l)$. And the item estimates can be updated accordingly until they converge within a small range.

A2. Computing the SEs

The second derivative of the log-marginalized likelihood with respect to any two parameters in item j, p_{jhc} and $p_{jh'c'}$, is

$$\frac{\partial^2 l(\mathbf{X})}{\partial p_{jhc}\partial p_{jh'c'}} = \sum\nolimits_{i=1}^{N}\left[\frac{1}{L(\mathbf{X}_i)}\frac{\partial^2 L(\mathbf{X}_i)}{\partial p_{jhc}\partial p_{jh'c'}} - \frac{1}{L^2(\mathbf{X}_i)}\frac{\partial L(\mathbf{X}_i)}{\partial p_{jhc}}\frac{\partial L(\mathbf{X}_i)}{\partial p_{jh'c'}}\right]. \tag{A14}$$

Note that the expected value of first term in (A14) disappears. With (A2) and (A7), one can derive

$$\frac{\partial L(\mathbf{X}_i)}{\partial p_{jhc}} = L(\mathbf{X}_i)\left(\frac{p(\boldsymbol{\eta}_{jh} \mid \mathbf{X}_i)X_{ijc}}{p_{jhc}} - \frac{p(\boldsymbol{\eta}_{jh} \mid \mathbf{X}_i)X_{ij0}}{p_{jh0}}\right). \tag{A15}$$

Accordingly, the expected value of (A14) becomes

$$-\sum_{i=1}^{N}\left(\frac{p(\boldsymbol{\eta}_{jh} \mid \mathbf{X}_i)X_{ijc}}{p_{jhc}} - \frac{p(\boldsymbol{\eta}_{jh} \mid \mathbf{X}_i)X_{ij0}}{p_{jh0}}\right)\left(\frac{p(\boldsymbol{\eta}_{jh'} \mid \mathbf{X}_i)X_{ijc'}}{p_{jh'c'}} - \frac{p(\boldsymbol{\eta}_{jh'} \mid \mathbf{X}_i)X_{ij0}}{p_{jh'0}}\right). \tag{A16}$$

(A16) gives the elements of the Fisher information matrix $\mathbf{I}(\mathbf{P}_j) = -E\left[\partial^2 l(\mathbf{X}) / \partial \mathbf{P}_j^{\ 2}\right]$, where $\mathbf{P}_j = \left\{ p_{jhc} \right\}$ including all $(C_j - 1)H_j$ independent parameters for item j (i.e., c > 0). Instead of computing the expectation, one can evaluate (A16) at $\hat{\mathbf{P}}_j = \left\{ \hat{p}_{jhc} \right\}$ with the observed data to obtain an approximate information matrix $\mathbf{I}(\hat{\boldsymbol{P}}_j)$. The square-root of the $(cH_j - H_j + h)$th diagonal element of $\mathbf{I}^{-1}(\hat{\boldsymbol{P}}_j)$ is $\mathrm{SE}[\hat{p}_{jhc}]$ approximately.
